# Recommendations to enhance breeding bird diversity in managed plantation forests determined using LiDAR

**DOI:** 10.1002/eap.2678

**Published:** 2022-08-03

**Authors:** Eleanor R. Tew, Greg J. Conway, Ian G. Henderson, David T. Milodowski, Tom Swinfield, William J. Sutherland

**Affiliations:** ^1^ Department of Zoology University of Cambridge, The David Attenborough Building Cambridge UK; ^2^ Forestry England Bristol UK; ^3^ British Trust for Ornithology Thetford UK; ^4^ School of GeoSciences University of Edinburgh Edinburgh UK; ^5^ National Centre for Earth Observation University of Edinburgh Edinburgh UK; ^6^ Department of Plant Sciences University of Cambridge Cambridge UK; ^7^ Biosecurity Research Initiative at St Catharine's (BIORISC), St Catharine's College University of Cambridge Cambridge UK

**Keywords:** breeding birds, forest management, forest structure, functional diversity, hierarchical partitioning, LiDAR, remote sensing, species diversity, taxonomic diversity

## Abstract

Widespread afforestation is a crucial component of climate mitigation strategies worldwide. This presents a significant opportunity for biodiversity conservation if forests are appropriately managed. Within forests, structural and habitat diversity are known to be critical for biodiversity but pragmatic management recommendations are lacking. We make a comprehensive assessment of the effects of habitat variables on bird populations using data from over 4000 ha of forested landscape. We combine high‐resolution remote sensing data with comprehensive management databases to classify habitat attributes and measure the response of six taxonomic and functional diversity metrics: species richness, Shannon diversity, functional richness, functional evenness, functional divergence, and functional dispersion. We use a novel approach that combines hierarchical partitioning analysis with linear models to determine the relative importance of different habitat variables for each bird diversity metric. The age class of forest stands was consistently the most important variable across all bird diversity metrics, outperforming other structural measures such as horizontal and vertical heterogeneity and canopy density. Shrub density and gap fraction were each significantly associated with one bird diversity metric. In contrast, variables describing within‐stand structural heterogeneity (vertical and horizontal) were generally less important while tree species identity (e.g., conifer or broadleaved) was not significant for any bird diversity metric. Each of the six bird diversity metrics had different patterns of independent variable importance and significance, emphasizing the need to consider multiple diversity metrics in biodiversity assessments. Similarly, the optimal resolution for remote sensing metrics varied between structural variables and bird diversity metrics, suggesting that the use of remote sensing data in biodiversity studies could be greatly improved by first exploring different resolutions and data aggregations. Based on the results from this comprehensive study, we recommend that managers focus on creating habitat diversity at the between‐, rather than exclusively within‐stand scale, such as by creating a matrix of different age classes, to maximize bird diversity. This recommendation for forest managers is powerful yet pragmatic in its simplicity.

## INTRODUCTION

Ambitious woodland planting has been pledged by many countries as a response to climate change (Popkin, 2019). For example, the United Kingdom (UK) government has committed to 30,000 ha of afforestation per year by 2025. This could yield considerable forest biodiversity conservation gains if afforestation takes place on land with otherwise low conservation value, such as degraded, low‐quality agricultural land. However, forest biodiversity is impacted by management strategies (Paillet et al., 2010; Penone et al., 2019), which directly affect forest structure and resource availability (Fuller, 2013; Fuller & Robles, 2018). For example, the size and age of trees, availability of deadwood, tree density, structural heterogeneity and tree species composition all have interrelated and important effects on a range of species (Fuller, 2013; Hilmers et al., 2018; Lindenmayer et al., 2006).

Forest plantations are often dismissed as “green deserts” (Horák et al., 2019). However, they have an important role to play in biodiversity conservation, particularly when they replace degraded land, where natural forests are scarce or where plantations can improve connectivity (Bremer & Farley, 2010; Brockerhoff et al., 2008; Graham et al., 2017; Pawson et al., 2013). Plantations can support a wide variety of taxa, including nationally important populations of species of conservation concern (Calladine et al., 2018; Graham et al., 2017). In general, forests managed for timber production have a lower species richness than unmanaged forests, but this result is highly variable, by both taxa and management intensity (Chaudhary et al., 2016; Paillet et al., 2010). For example, in the UK, a lack of sustainable woodland management is one of the leading causes of biodiversity decline (Hayhow et al., 2019). Delivering high biodiversity value is compatible with both timber production and carbon storage objectives (Asbeck et al., 2021; Schall et al., 2020).

Structural complexity of plantations is a key factor influencing species diversity and abundance, therefore management strategies can have a profound impact (Humphrey et al., 2015; Nájera & Simonetti, 2010). For example, continuous‐cover (uneven‐aged) forestry systems (where trees are harvested singly or in small patches) create greater structural heterogeneity at the small scale than conventional clearfell management systems (where larger areas are felled at the same time). It is often assumed that increasing within‐compartment diversity will benefit biodiversity; although this is supported by some individual studies (e.g., Calladine et al., 2015; Peura et al., 2018), the overall evidence for this is mixed and weak (Bus de Warnaffe & Deconchat, 2008; Farwig et al., 2017; Schall et al., 2018). A recent review of global studies found the claim that uneven‐aged silvicultural regimes are better for ecological diversity and processes than even‐aged silviculture was unsubstantiated (Nolet et al., 2018). To maximize the biodiversity potential of new afforestation, and to increase the value of existing woodlands, we need to better understand the relationships between forest management and biodiversity in different situations.

Birds are widely used as indicators of forest biodiversity (Alexander et al., 2017; Augustynczik et al., 2019). In Europe, birds are the most monitored taxonomic group, yielding extensive and regularly updated data sets (Eglington et al., 2012; Schmeller et al., 2012). Birds can be effective indicators of a wide variety of taxa, although results are mixed and context‐specific (Eglington et al., 2012; Gao et al., 2015). Despite a large body of literature focusing on the habitat requirements of forest birds, knowledge gaps remain. For example, forest management is rapidly changing in response to unprecedented challenges, including climate change and disease (Messier et al., 2015; Potter & Urquhart, 2017), leading to novel forest systems involving new species, mixtures, and silvicultural systems (including more continuous‐cover systems). The effects on forest birds, and biodiversity more widely, is poorly understood (Fuller & Robles, 2018; Schall et al., 2018).

In addition to the quality of forest habitat, which is largely determined by its structure and floristic components, a variety of factors influence bird community assemblages and diversity (Fuller & Robles, 2018; Hewson et al., 2011). For example, climate, surrounding land uses, regional bird population sizes and interspecific interactions all have interrelated effects (Calladine et al., 2018; Goetz et al., 2014). To date, most studies exploring management effects on bird diversity in temperate forests come from North America; evidence and understanding in Europe is lacking and, given intercontinental differences, results cannot necessarily be extrapolated (Fuller, 2013; Fuller & Robles, 2018). In particular, predominantly coniferous lowland plantations have received little attention.

Management decisions take place at the scale of forest stands, which are discrete areas with the same tree species composition and structure (Forestry Commission, 2017). Forest stands are also an appropriate scale to study the habitat requirements of most bird species because resource use and interactions between individuals tend to occur at relatively small scales (Cushman & McGarigal, 2004; Hewson et al., 2011). However, habitat features and structure are often considered to be more important determinants of community assemblages and diversity than broad descriptions of management type (Müller et al., 2010; Penone et al., 2019). Using remote sensing technologies, we can now measure habitat attributes in fine detail over large areas (Garabedian et al., 2017). This presents an opportunity to improve our understanding of how forest management affects biodiversity by determining how management creates, and species respond to, subtle differences in habitat.

The detailed habitat information generated from remote sensing also allows us to explore how species perceive and respond to structure at different spatial scales. We know that species respond to different structural characteristics at different scales, yet most ecological studies do not explicitly incorporate this in analysis (Mcgarigal et al., 2016). However, scale optimization (testing different scales of environmental variables to find the scale at which species respond most strongly) can be achieved relatively easily by grouping remote sensing data into different resolutions. This delivers important improvements to ecological analysis.

The majority of studies exploring the effects of forest management on biodiversity use metrics of taxonomic diversity, most notably species richness (Chaudhary et al., 2016; Lelli et al., 2019; Paillet et al., 2010). However, species richness does not fully capture the multiple components of ecological communities and can even be misleading because different diversity metrics often show contrasting responses to habitat attributes (Lyashevska & Farnsworth, 2012; Penone et al., 2019). In addition to taxonomic metrics (such as species richness and Shannon diversity), functional diversity metrics are particularly useful as they capture the diversity of species' traits, which can be related more directly to ecosystem functions and processes (Cadotte et al., 2011; Hatfield et al., 2018; Petchey & Gaston, 2006). This is relevant for understanding ecosystem productivity and stability and informing conservation strategies (Lelli et al., 2019; Schleuter et al., 2010), particularly as functional diversity can be more sensitive to disturbance than taxonomic diversity (Cadotte et al., 2011; Flynn et al., 2009; Mao et al., 2021). However, the relationships between taxonomic and functional diversity metrics are unresolved for many systems, so a combined approach is useful (Mao et al., 2021; Parker et al., 2018; Villéger et al., 2010).

Functional diversity is commonly represented by three independent metrics: functional richness, functional evenness, and functional divergence (Mason et al., 2005; Mouchet et al., 2010; Villéger et al., 2008). More recently, functional dispersion has also been proposed as a useful complement to these measures (Laliberté & Legendre, 2010). Functional richness and evenness are counterparts to species richness and evenness. Functional “richnesss” is a measure of the range of traits present in a community, which exploit different environmental niches (Mason et al., 2005). Low functional richness implies that there are few niches available or that elements of the community are missing. Under changing environmental conditions, communities with greater functional richness are expected to have higher resilience as there will already be traits present that can adapt to and take advantage of the new conditions. The distribution of abundances across these different traits is captured by functional “evenness.” Even in communities with high functional richness, there may be low functional evenness if the majority of species and individuals have similar traits and only a few individuals have different traits. Low functional evenness in a community implies that the distribution of species across the traits is uneven and therefore some elements of niche space may be underutilized, potentially decreasing ecosystem productivity and resilience (Mason et al., 2005). The differentiation between groups of traits (or niches) is represented by functional “divergence.” Communities with high functional divergence have lower resource competition between species and therefore are expected to use resources more efficiently, with overall higher ecosystem function. Finally, functional “dispersion” measures the distribution of species' traits weighted by abundance; it is an evolution of functional richness, accounting for the fact that extreme trait values with low abundance would skew the richness metric. High functional dispersion indicates that the dominant (i.e., most abundant) species have complementary, rather than overlapping, trait niches (Mason et al., 2012). Similarly to functional richness, communities with high functional dispersion support a greater range of ecological functions and buffer ecosystems undergoing environmental change through a diversity of responses (Cooke et al., 2019). However, functional dispersion may reveal changes in community composition that functional richness cannot detect, for example if environmental change reduces the abundance of some species but does not cause extinctions (Valdivia et al., 2017).

In this study, we explore how management strategies can maximize the benefits of commercial forest plantations for bird diversity. We analyze the relationship between habitat attributes and bird diversity, using six taxonomic and functional metrics: species richness, Shannon diversity, functional richness, functional evenness, functional divergence and functional dispersion, to answer three interrelated questions. First, what is the relative importance of different habitat attributes for bird diversity, and what does this mean for management? Second, do these relationships vary for the different bird diversity metrics, and in what way? Finally, what spatial resolution (granularity) of habitat or structural data is most informative for explaining bird diversity? We use these results to make recommendations for the management of forest plantations.

## METHODS

### Study site

Thetford Forest is a large plantation (18,535 ha) in East Anglia, UK. In broad structure, it is typical of many commercial forests globally. Two‐thirds of the forest is coniferous, of which around 80% is monoculture. The remaining area comprises broadleaved and mixed woodland and open heathland. The predominant timber crop species are Corsican pine *Pinus nigra* and Scots pine *Pinus sylvestris*. Most of the productive forest area is managed through rotational clear‐cutting (with cutting at around 50–60 years), although there are some areas of continuous‐cover management. To facilitate management planning, the forest is divided into compartments (equivalent to stands), which are delineated based on their species composition, tree ages and management prescription. Forest compartments have a mean size of 2.9 ha, although there are a variety of sizes (median 1.7 ha, interquartile range 3.2 ha); 81% of compartments are under 5 ha.

The forest is one of the publicly owned state forests managed by Forestry England. The majority of the forest was planted following the First World War as part of an effort by the UK government to establish a strategic timber reserve. Large areas of marginal agricultural land were afforested between 1922 and 1950 (Dannatt, 1996). The habitats surrounding the forest are a mixture of agriculture and lowland heath.

Thetford Forest is an important biodiversity site, being designated as a Special Protection Area (SPA) for its breeding populations of Nightjar *Caprimulgus europeaus* and Woodlark *Lullula arborea*, and a Site of Special Scientific Interest for both birds together with its invertebrate and plant assemblage (Natural England, 2000). Nightjar and woodlark nest in the early successional stages of commercial planting, and thus the SPA designation requires a large proportion of the forest to be managed with rotational clear‐cutting.

### Bird and spatial data

**FIGURE 1:**
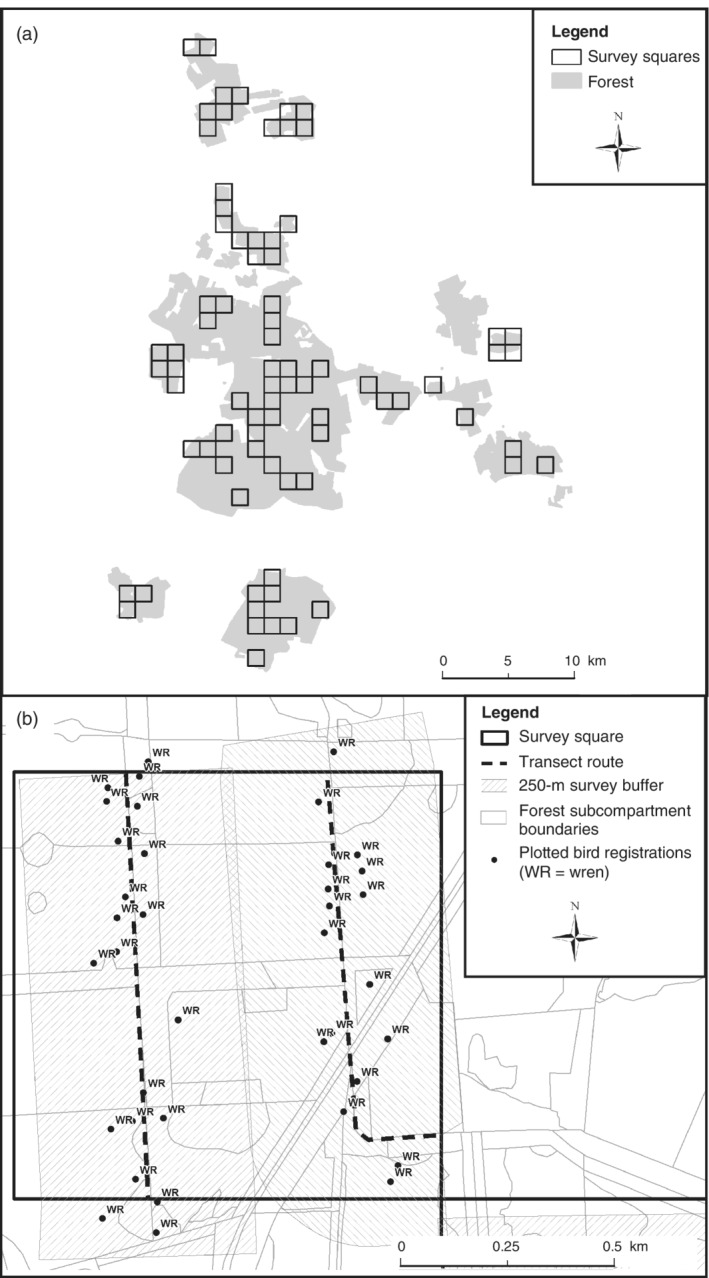
(a) The 1‐km squares included in the breeding bird surveys. (b) Example of transect routes and mapped registrations of one species (wren) within a 1‐km survey square. Two 1‐km transects were walked within each square (dashed lines), with a 250‐m buffer on both sides of the transect (hashed area), to give maximum coverage of the square. Bird observations were plotted on 1:2500 scale map (black points).

Breeding bird surveys took place between 17th April and 14th June in 2015 and 2017, following the BTO/JNCC/RSPB Breeding Bird Survey methodology (Harris et al., 2019) with additional mapped bird registrations. One‐square‐kilometer survey squares aligned to the British National Grid were selected to provide representative coverage of the forest (Figure 1a), including compartment size and soil type. Two 1‐km transects were walked in each 1‐km^2^ square between 06:00 and 11:00. The observer moved at a steady pace recording all birds seen or heard within 250 m either side of the transect. Transect routes were planned to give maximum coverage of the 1‐km^2^ square, while following existing tracks and paths where possible. Data were plotted on a 1:2500 scale map showing forest compartment boundaries and tree species/land use, to allow accurate association of birds with habitats (Figure 1b). Each 1‐km^2^ square was visited twice, at least two weeks apart. The data were grouped by forest compartment, and the maximum bird count per species from the two visits was used for analysis. Using the maximum count is the standard statistical approach for these two‐visit surveys, accounting for varying levels of activity between species (e.g., resident and early arriving migrants have increased activity in the early part of the breeding season, whereas late arriving migrants have peak activity in the later part of the season; Harris et al., 2019).

Each forest compartment is a unit of forest management with detailed, long‐term records, including tree species composition and age classes. Remote‐sensing data were available for only part of the forest in the two survey years, so we retained only the corresponding 1‐km^2^ bird survey squares for analysis. We clipped compartment shapefiles to the edges of the survey area (250 m from each transect line) for analysis of forest structural data. Compartments were excluded if management information was missing or if the survey area was under 0.05 ha.

### Remote‐sensing data

We used airborne Light Detection and Ranging (LiDAR) remote sensing data collected in March 2015 and 2017 to quantify structural attributes for each forest compartment. LiDAR generates a point cloud of ground, vegetation, and object heights using a laser scanning technique. The data were acquired using a fixed wing platform (2015, Riegl Q680; 2017, Optech Orion) at an elevation of 2000–2300 feet. The mean point density was 24 points/m^2^. Different areas of the forest were covered by the LiDAR in each year with no overlap; however, this was simply a practical data collection limitation and there was no fundamental difference in the habitat types covered between the 2 years' data collection.

Firstly, we produced a digital elevation model by classifying point returns as ground or non‐ground. Point heights were then normalized with respect to the digital elevation model, from which a pit‐free canopy height model was produced at a resolution of 0.5 m (Khosravipour et al., 2014). All initial processing used LAStools algorithms (Isenburg, 2018). Secondly, we produced a vertical plant area distribution profile at a resolution of 10 m. This approach is based on the MacArthur‐Horn method that was developed to estimate foliage profiles in the field (MacArthur & Horn, 1969), and adapted for use with LiDAR data (Hopkinson et al., 2013; Milodowski et al., 2021). Appendix S1 gives further details and graphical illustrations of the plant area distribution profiles for different stands.

From the canopy height model and plant area distribution profile, we derived six metrics to describe the habitat structure: top canopy height, gap fraction, horizontal heterogeneity, vertical evenness, shrub density and canopy density (Table 1). By taking the mean pixel value, we aggregated the canopy height model into four different spatial resolutions: 0.5, 2, 5, and 10 m, and extracted the canopy height model pixels for each compartment at each resolution. Similarly, we separated the plant area distribution profile by compartment and calculated the mean value of each vertical segment. Calculations of structural metrics are shown in Table 1.

**TABLE 1:** Structural metrics calculated from the LiDAR data.

Structural metric	Processed LiDAR data used	Resolution(s) (m)	Calculation for each compartment
Top canopy height	Canopy height model	0.5, 2, 5, 10	Mean pixel value
Gap fraction	Canopy height model	0.5, 2, 5, 10	Percentage of pixels that were less than two‐thirds of the mean top canopy height value
Horizontal heterogeneity	Canopy height model	0.5, 2, 5, 10	Moran's *I* index of all pixel values
Vertical evenness	Plant area distribution values	10	Pielou's evenness index (Shannon index of plant area distribution values divided by the logarithm of the number of plant area distribution segments)
Shrub density	Plant area distribution values	10	Sum of plant area distribution values in vertical segments above 1 m and below 5 m in height
Canopy density	Plant area distribution values	10	Sum of plant area distribution values in vertical segments in the top third of the canopy. To account for within‐stand variability, the top of the canopy was taken as the mean top canopy height + 2 standard deviations. Vertical segments entirely within the top third of this value were classified as the canopy

### Compartment grouping

The sorted data contained 1476 compartments from 51 1‐km^2^ survey squares in 2015 and 399 compartments from 16 1‐km survey squares in 2017, covering a total area of 4404 ha. It was impossible to sensibly calculate species diversity metrics for individual compartments as their small size yielded generally few individual birds. Therefore, we summarized data across compartments to compare bird community assemblages in different habitat types. First, we used the detailed management database to determine each compartment's management type and age class (planting years were used to calculate the age of trees in each compartment; Table 2). We then grouped compartments into unique combinations of management type and age class. Finally, we excluded groups with fewer than 10 compartments (due to low replication and the small total area of these groups), leaving 31 groups of 1752 compartments covering a total area of 4217 ha. We calculated the mean maximum count for each bird species across the compartments in each group and divided this by a species‐specific detectability constant to account for detectability differences between bird species. The detectability constants we used were calculated for birds at 100 m, using UK‐wide Breeding Bird Survey data (i.e., the same survey techniques used in this study; Johnston et al., 2014).

**TABLE 2:** Forest management type and age class definitions.

Detailed category	Simplified category
Management type	
Corsican pine *Pinus nigra* monoculture	Conifer
Scots pine *Pinus sylvestris* monoculture	Conifer
Douglas fir *Pseudotsuga menziesii* monoculture	Conifer
“Other” conifer monoculture	Conifer
Birch *Betula* spp. monoculture	Broadleaved
“Other” broadleaved monoculture	Broadleaved
Conifer and broadleaved mixture (largest species component is conifer)	Mixture
Conifer and broadleaved mixture (largest species component is broadleaved)	Mixture
Open	Open/other
Open with trees	Open/other
Other	Open/other
Felled	Open/other
Age class	
Restock (0–6 years)	Restock/pre‐thicket
Pre‐thicket (7–11 years)	Restock/pre‐thicket
Thicket (12–21 years)	Thicket/pole
Pole (22–45 years)	Thicket/pole
Mature (46 years +)	Mature
Mixed ages	Mixed
Open/other	Not applicable

*Note*: The “simplified category” column shows the groups used in the final analysis.

For each management/age class group we summarized the structural data calculated from the LiDAR by taking the mean values of all the compartments in each group. The total area of compartments varied between the different groups; we therefore transformed the total area data using a logarithmic function and included this as a variable in our analysis to account for its potentially confounding effects (such as the expected species–area relationship for species richness and diverse relationships with functional diversity metrics; Karadimou et al., 2016; MacArthur & Wilson, 1963). To increase the number of groups in each category used in the statistical analysis to make it more robust, we then simplified the management type categories into conifer, broadleaved, mixture (i.e., conifer and broadleaved) and open/other; age class categories were restock/pre‐thicket, thicket/pole, mature, mixed ages, and not applicable (i.e., due to being open/other management type; Table 2). This gave us 31 compartment groups in total, each of which became an independent data point used in the subsequent statistical analysis, with the values for each group underpinned by an extensive underlying data set (Appendix S2 details the number of compartments and total area in each group).

### Statistical analysis

For the bird communities in each of the 31 compartment groups, we calculated the two taxonomic diversity metrics (species richness and Shannon diversity) using the *vegan* R package (Oksanen et al., 2018), and the four functional diversity metrics (functional richness, functional evenness, functional divergence, and functional dispersion) using the *FD* R package (Laliberté et al., 2014). For the functional diversity metrics, we included species‐specific trait information on body mass, migratory status, nest site, diet, and foraging strata (Appendix S3: Table S1). Please see Appendix S4 for more detail on exactly how the functional diversity metrics were calculated statistically, including a graphical visualization. We tested the normality of each metric using the Shapiro‐Wilk test. Where appropriate, we normalized the distributions using Box‐Cox transformations, finding the best lambda parameter using profile log‐likelihoods (using the *MASS* R package; Venables & Ripley, 2002).

We used a novel statistical approach combining hierarchical partitioning analysis with linear models to determine the relative importance of different habitat and structural variables in predicting each bird diversity metric. As is often the case in ecological studies, many of the habitat variables were colinear with one another, so we needed to first select a group of independent variables to build a linear model. Hierarchical partitioning allowed us to select the best combination of independent habitat variables in an objective and statistically robust way, rather than making a subjective choice of variables based on a priori assumptions.

**FIGURE 2:**
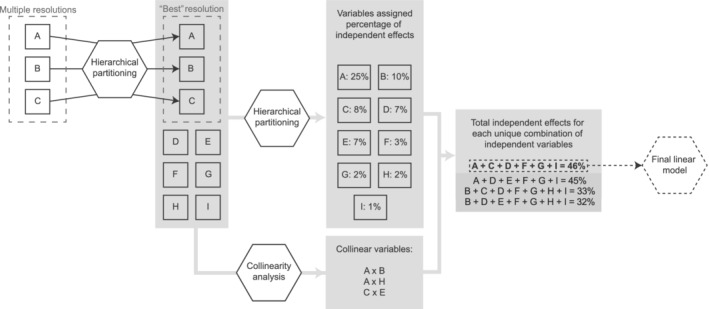
Flow diagram showing selection of variables in the statistical analysis. Hexagons indicate different types of analysis, gray boxes indicate results from previous analytical stages. The letters indicate different variables. Variables A, B, and C correspond to top canopy height, gap fraction and horizontal heteorogeneity, which were calculated at multiple resolutions. Values given are demonstrative only and do not correspond to results from this paper.

We first used collinearity analysis to determine which variables were correlated with one another. For each of the six diversity metrics, we then used hierarchical partitioning to attribute model variation to each habitat variable, and combined this information with the collinearity results to find the combination of variables that were independent and had the highest total contribution to model variance. Figure 2 outlines the process; more detail about the individual steps are as follows.

#### Collinearity analysis

We checked for collinearity of variables (such as age class and top canopy height) by running pairwise correlation tests between different variables. Following conventional practice, we set a collinearity threshold of |0.7|, and determined which variables were independent (see Appendix S5 for results).

#### Hierarchical partitioning

We used hierarchical partitioning to determine the relative importance of different variables in predicting each of the diversity metrics. Hierarchical partitioning attributes goodness of fit measures for multivariate regression to individual variables, through considering all possible models with all combinations of variables (Chevan & Sutherland, 1991). It averages the influence of each variable in all models in which it could feature. For example, if there are potential variables A, B, and C, it evaluates the influence of A in models A, AB, AC, and ABC (through pairwise comparisons with models without variable A, e.g., comparing ABC with BC, AC with C, etc.), and takes the average (Chevan & Sutherland, 1991; MacNally, 2000). The variation for each variable is partitioned into the independent contribution (the influence of the variable independent of all other variables) and joint contribution (the influence of the variable in combination with the other variables). It is an attractive analytical technique because it can deal with multicollinearity between independent variables (Olea et al., 2010). We used the *hier.part* package in R and limited the number of variables to nine, in line with package recommendations (Olea et al., 2010; Walsh & MacNally, 2013).

First, we reviewed the spatial resolution of structural variables. Top canopy height, gap fraction and horizontal heterogeneity were all calculated at a range of resolutions (0.5, 2, 5, and 10 m) and we used hierarchical partitioning to select the “best” resolution, i.e., that with the highest percentage of independent effects for each combination of diversity metric and variable, using *R* ^2^ as the goodness of fit measurement (Figure 2). In addition to these three structural variables, we then also included the habitat variables broad management type, age class, vertical evenness, shrub density, and canopy density. Finally, as each compartment group had different total areas, (log) total area was included to account for the potentially confounding effect of area on diversity metrics. The final hierarchical partitioning analysis therefore included a total of nine variables for each diversity metric.

#### Linear model

We selected those variables that were independent and had the greatest total percentage of independent effects (calculated from the hierarchical partitioning) for each of the six diversity metrics (Figure 2). We built a separate linear regression model including this selected subset of variables for each diversity metric and checked the model fit using the *DHARMa* R package (Hartig, 2018). We assessed the overall model significance and the significance of each variable using a type 2 ANOVA (which tests the effects of each variable after all other variables are included, so variable order is not important, in contrast to a type 1 ANOVA that tests variables sequentially) using the *car* R package (Fox & Weisberg, 2011). As well as determining the overall significance of the variable, we compared the categorical variable broad management type to a baseline of “conifer” and age class to a baseline of “mixed ages.”

All analysis was performed in R (R Core Team, 2018). The data supporting this study are available from the University of Cambridge repository service Apollo at the following doi: https://doi.org/10.17863/CAM.83342 (Tew et al., 2022).

## RESULTS

### Spatial and structural data

**FIGURE 3:**
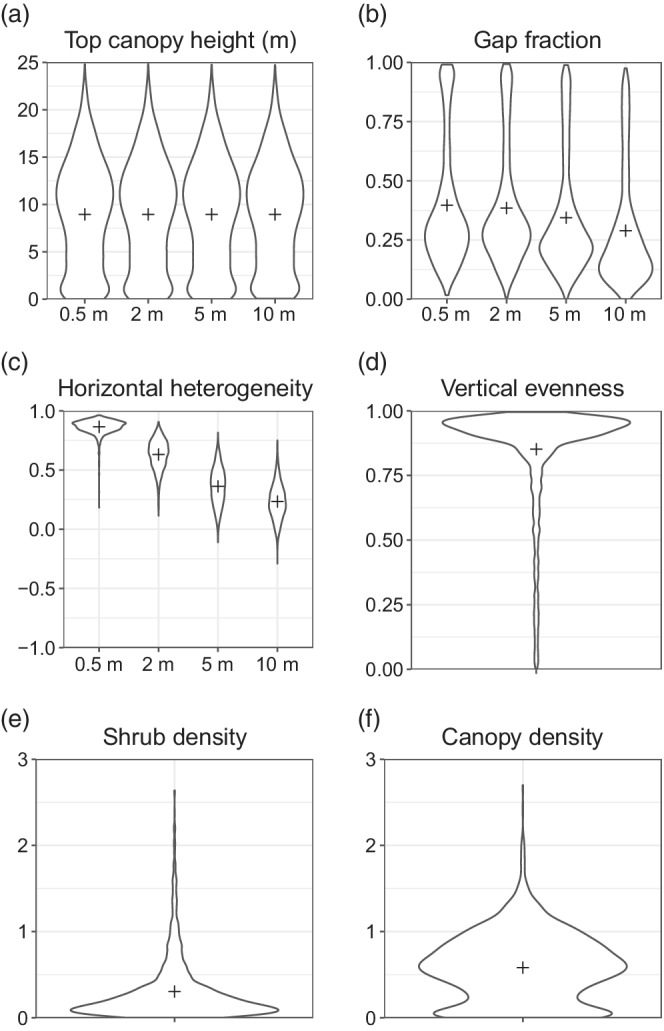
The variation in structural characteristics derived from the LiDAR data. The violin plots show the variation across all compartments. Crosses indicate the means. The structural metrics shown in parts (a)–(c) were calculated at different resolutions. The structural metrics shown in parts (d)–(f) were derived from the plant area distribution values at one resolution (10 m). Note that the *y*‐axis differs across the six panels.

The mean compartment area was 2.35 ha (±2.53 SD), ranging from 0.053 to 15.77 ha. Mean top canopy height was 8.95 m, with a maximum height of 24.79 m. The four different LiDAR resolutions tested (0.5, 2, 5, and 10 m) produced very similar values for top canopy height (means all rounding to 8.95 m, standard deviation ranging from 5.53 to 5.60) as they were aggregating means at different levels (Figure 3a). The mean gap fraction and horizontal heterogeneity decreased with increasing LiDAR resolution (Figure 3b,c). This effect arises because as granularity increases, pixels average across fine‐scale heterogeneities, and smaller canopy gaps are no longer resolved. For gap fraction, the mean value decreased from 0.40 (0.5 m resolution) to 0.29 (10 m resolution), although standard deviation was similar across all resolutions (range 0.24–0.26). The mean value for horizontal heterogeneity decreased more dramatically, from 0.87 (0.5 m resolution) to 0.23 (10 m resolution); standard deviation also increased with coarser resolutions (from 0.06 for 0.5 m resolution to 0.16 for 5 m resolution and 0.15 for 10 m resolution). The majority of compartments had high vertical evenness (mean = 0.85) although a full range of values was observed (Figure 3d). The average values for shrub density (0.30) and canopy density (0.58) were low across compartments although there was higher variation for canopy density (SD = 0.39; Figure 3e,f).

### Bird observations

In the 1752 compartments used in the final analysis, 48 bird species were recorded, of which 35 species had more than 25 observations and 17 species had over 100 observations. The mean number of observations was 158.2. Wren *Troglodytes troglodytes* was the most common species (1224 observations), with almost twice the number of observations as the next most common species (robin *Erithacus rubecula*: 641 observations). Appendix S6: Figure S1 provides further details.

### Hierarchical partitioning

#### Variable spatial resolutions

We found that the optimal LiDAR resolution for each structural variable (top canopy height, gap fraction, horizontal heterogeneity) depended on the diversity metric (Appendix S7). The optimal resolutions for bird functional divergence and functional dispersion were 0.5 m for all the structural variables. However, the optimal resolutions for bird species richness, Shannon diversity, functional richness, and functional evenness were all 5 or 10 m (with the exception of gap fraction for Shannon diversity, for which the optimal resolution was 0.5 m). The 2‐m resolution was not the optimal resolution for any structural metric. For bird species richness, functional richness and functional evenness, the optimal resolution for horizontal heterogeneity was coarser (10 m) than for top canopy height or gap fraction (5 m; with the exception of gap fraction for functional evenness, which was 10 m).

#### Final variables

In the hierarchical partitioning for all nine variables (using the “best” variable resolution where applicable), total *R* ^2^ values were between 0.77 (functional divergence) and 0.95 (functional evenness; Table 3). The quality of functional richness given by the dbFD function (*FD* R package) was 0.95 (which can be interpreted as a *R* ^2^‐like ratio).

**TABLE 3:** *R* ^2^ values generated by the hierarchical partitioning analysis (showing values when all nine variables were included and when only the final selected variables were included) and the final linear models.

Diversity metric	Hierarchical partitioning analysis		Final linear model analysis	
	*R* ^2^ from all variables	*R* ^2^ from selected variables	Final model *R* ^2^	Final model adjusted *R* ^2^
Species richness	0.94	0.86	0.93	0.89
Shannon diversity	0.84	0.69	0.74	0.62
Functional richness	0.88	0.82	0.85	0.78
Functional evenness	0.95	0.68	0.93	0.90
Functional divergence	0.77	0.28	0.65	0.52
Functional dispersion	0.85	0.35	0.73	0.65

**FIGURE 4:**
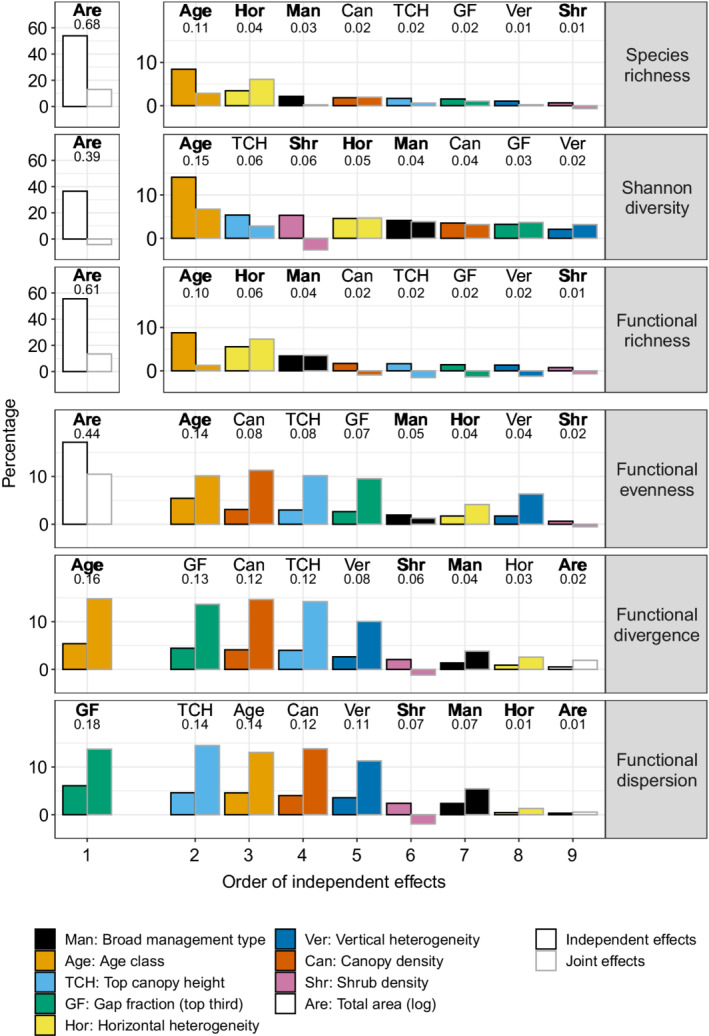
Hierarchical partitioning of the final nine variables for each of the six diversity metrics. Bars show the percentage of independent and joint effects attributed to each variable. Variables are ordered by the rank of independent effects. For clarity, the area variables for the top three panels are shown on a separate *y*‐axis scale; all other variables are on the same scale. Bars are color coded by variable, and three‐letter codes above bars aid interpretation (see key). Note that “Age” is a categorical variable, not continuous (see Table 2). Values above each bar are the absolute values of independent effects, i.e., the *R* ^2^ attributed to each variable. The variables selected for the final linear models are indicated by bold font of the labels above the bars.

For species richness, Shannon diversity, functional richness, and functional evenness, total area had the greatest percentage of independent effects, followed by age class (Figure 4). Total area was attributed by far the greatest proportion of *R* ^2^, particularly for species richness (*R* ^2^ = 0.68) and functional richness (*R* ^2^ = 0.61). In contrast, for functional divergence and functional dispersion, area was the least important variable (*R* ^2^ = 0.02 and 0.01, respectively). The relative importance of variables (i.e., the pattern of variables ordered by *R* ^2^) was different for each diversity metric, but age class was universally important across all diversity metrics (Figure 4). The variable order and values of effect size were similar for species richness and functional richness (total area > age class > horizontal heterogeneity > broad management type > canopy density > top canopy height > gap fraction > vertical heterogeneity > shrub density), and they were also similar for the last five variables for functional divergence and functional dispersion (vertical heterogeneity > shrub density > broad management type > horizontal heterogeneity > total area), indicating that each of these pairs of diversity metrics were influenced in the same way by the structural variables (Figure 4).

The subset of independent variables carried forward into the final linear models were total area, age class, horizontal heterogeneity (except for functional divergence), broad management type and shrub density. For functional dispersion, gap fraction was chosen instead of age class. (Gap fraction and age class were collinear so could not both be included in the final linear model [see Appendix S5]. Age class was attributed the greater proportion of independent effects from the hierarchical portioning—and therefore included in the final linear model—for all the diversity metrics except functional dispersion, where gap fraction had a higher percentage of independent effects and so was included instead, Figure 4.) The main factor controlling the selection of variables for the final models was whether they were collinear with age class or gap fraction, which excluded canopy density, top canopy height and vertical evenness. The *R* ^2^ of these final models remained high, ranging from 0.65 (functional divergence) to 0.93 (species richness, functional evenness), with adjusted *R* ^2^ values between 0.52 (functional dispersion) and 0.90 (functional evenness; Table 3).

### Linear model outputs

**FIGURE 5:**
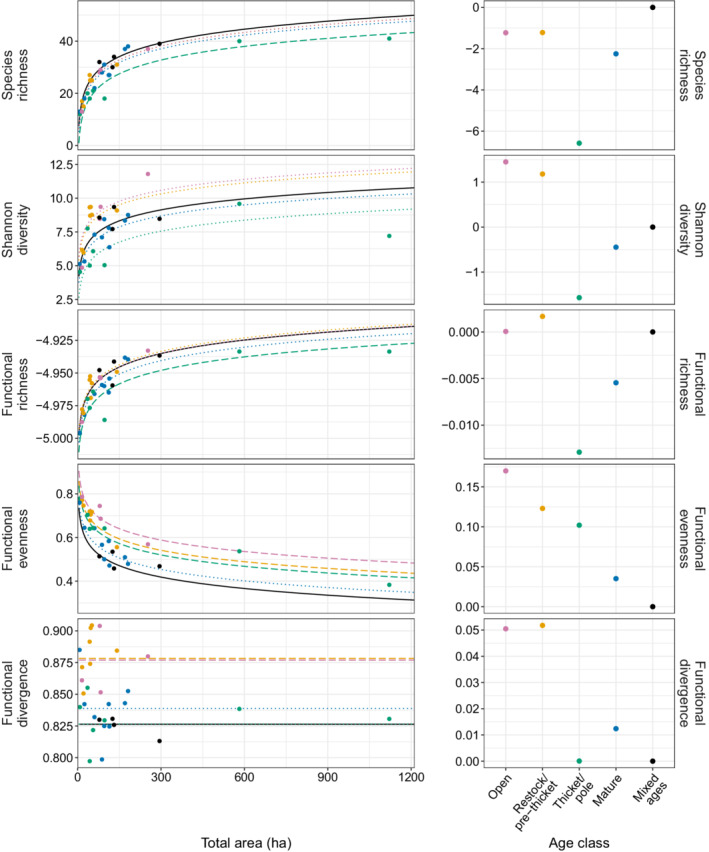
Significant linear relationships between (log) total area and diversity metrics. Panels on the left show the raw data (points) and modeled relationship (lines), plotted for non‐transformed area values to aid interpretation. Line type shows whether the age class is significantly different to the reference “mixed” age class: dashed, significantly different from “mixed”; dotted, not significantly different from mixed. Graphs on the right show the intercept estimate for the different age classes, relative to the reference mixed age class (value of 0). Colors indicate the different age classes: pink, open/other; yellow, restock/pre‐thicket; green, thicket/pole; blue, mature; black, mixed ages.

The full linear model was highly significant for each diversity metric (all *p* < 0.001; Table 4). Age class was significant in all models (except functional dispersion, where it was not included). Compared to the mixed age class, the thicket/pole age class had significantly lower values of species richness and functional richness, and significantly higher values of functional evenness. Additionally, restock/pre‐thicket and open/other categories had significantly higher values of functional evenness and functional divergence (Figure 5). Comparing the intercept estimates across all the diversity metrics, as a general pattern, bird diversity was highest in the open space or early successional stages but decreased in the intermediate thicket/pole age class (Table 4, right‐hand column in Figure 5). With the exception of functional evenness, diversity increased again in the mature age class but not to the same high levels at the earlier successional stages.

**TABLE 4:** ANOVA results (effect estimates, standard errors and significance values) of the final linear models for each diversity metric.

	Species richness		Shannon diversity		Functional richness		Functional evenness		Functional divergence		Functional dispersion	
	*p* = 6.88e−10 (***)		*p* = 2.10e−04 (***)		*p* = 9.91e−07 (***)		*p* = 3.9e−10 (***)		*p* = 0.0011 (***)		*p* = 2.95e−05 (***)	
Variable	Estimate (SE)	*p*	Estimate (SE)	*p*	Estimate (SE)	*p*	Estimate (SE)	*p*	Estimate (SE)	*p*	Estimate (SE)	*p*
(Intercept)	−2.30 (3.97)		1.23 (1.85)		−5.02 (0.012)	***	0.87 (0.051)	***	0.83 (0.025)	***	0.13 (0.084)	
Total area (log)	7.58 (0.57)	***	1.17 (0.21)	***	0.015 (0.0018)	***	−0.075 (0.0073)	***	−0.0018 (0.0039)		0.0011 (0.0020)	
Horizontal heterogeneity	−11.90 (14.00)		1.75 (4.67)		0.019 (0.044)		−0.12 (0.18)		N/A		0.095 (0.092)	
Age class		**		**		*		***		**	N/A	
Mixed ages												
Restock/pre‐thicket	−1.22 (2.11)		1.18 (0.83)		0.0017 (0.0065)		0.12 (0.027)	***	0.052 (0.016)	**		
Thicket/pole	−6.58 (1.90)	**	−1.57 (0.78)	^†^	−0.013 (0.0059)	*	0.10 (0.024)	***	4.80e−05 (0.014)			
Mature	−2.25 (1.75)		−0.45 (0.69)		−0.0055 (0.0055)		0.035 (0.023)		0.012 (0.013)			
*Open/other*	−*1.23* *(2.28)*		*1.45* *(0.95)*		*5.17e−05* *(0.0071)*		*0.17* *(0.029)*	***	*0.051* *(0.017)*	*		
Management type								^†^				
Conifer												
Mixture	−0.26 (1.48)		0.24 (0.63)		−0.0018 (0.0046)		0.045 (0.019)	*	−0.0011 (0.011)		0.0016 (0.0058)	
Broadleaved	3.37 (1.72)	^†^	0.83 (0.79)		0.0026 (0.0053)		−0.0051 (0.022)		3.06e−04 (0.013)		−0.0030 (0.0069)	
*Open/other*	−*1.23* *(2.28)*		*1.45* *(0.95)*		*5.17e−5* *(0.0071)*		*0.17* *(0.029)*	***	*0.051* *(0.017)*	*	−0.0021 (0.0081)	
Shrub density	3.65 (2.3)		1.88 (0.91)	^†^	4.18e−4 (0.0072)		0.015 (0.030)		0.024 (0.016)		0.029 (0.0091)	**
Gap fraction (top third)	N/A										0.070 (0.012)	***

*Note*: ***p < 0.001; **p < 0.01; *p < 0.05; ^†^ *p* < 0.1. The *p* value of the full linear model is given under the respective headings. The “open/other” category is equivalent in both age class and management type categories; they cannot be included in the same model and depending on variable order, one category is removed. Values for both categories are identical and are shown in italics. The “mixed ages” category is the reference categorical level for age class and the “conifer” category is the reference categorical level for management type, hence they are left blank. N/A indicates variables not included in the final model for that diversity metric. SD, standard error.

Total area was a significant predictor for all dependent variables except functional divergence and functional dispersion (Table 4). Species richness, Shannon diversity and functional richness increased with total area, while functional evenness decreased with total area (Table 4 and Figure 5).

**FIGURE 6:**
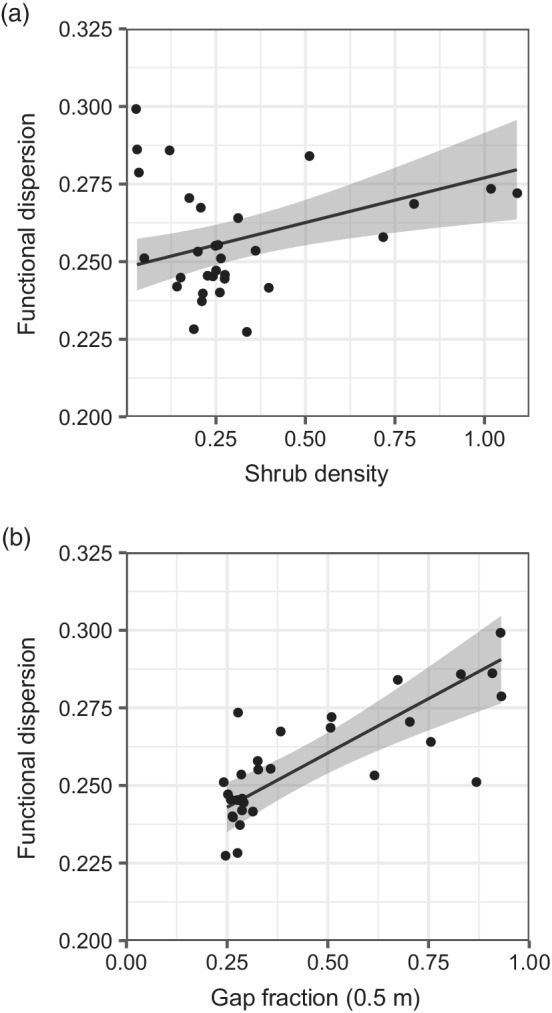
Significant linear relationships between functional dispersion and (a) shrub density and (b) gap fraction. The black line indicates the regression line and the gray shading indicates the 95% confidence interval.

Shrub density was significantly associated with functional dispersion, although the effect size (0.03) was marginal over the range of values observed in the forest (0.02–1.09; Table 4 and Figure 6). Gap fraction was also a significant predictor of functional dispersion and had a greater effect on predicted values (0.07, Figure 6). Although widely included in the final models, neither horizontal heterogeneity nor management type was significantly associated with any of the diversity metrics (Table 4).

## DISCUSSION

The objective of this study was to determine the relative importance of different habitat attributes for bird diversity in a commercial forest plantation, and to relate this to management. We show that compartment area and age class (which included mixed ages and open/other categories, in addition to descriptors of even‐aged structure) were the predominant factors affecting bird diversity. Individual structural variables tended to be collinear with age class and explain less variation in the hierarchical partitioning models. Their small additional effect was further emphasized by the relatively small decrease in model *R* ^2^ when they were dropped from the final models (Table 3). Of course, this does not mean that structure is unimportant, after all, age class is simply a way of defining different structural categories; however, in this study, age class was found to be the most consistent metric predicting bird diversity.

Stand age is widely reported to be a fundamental variable affecting forest bird assemblages and diversity (Moning & Müller, 2009; Reise et al., 2019). We deliberately matched our age class categorizations to those used by forest practitioners, so our findings could be interpreted and applied practically. Our results support the general observation that early and late successional stages (e.g., pre‐thicket and mature age classes) tend to have the highest diversity, with fewer species associated with intermediate stages (e.g., thicket and pole age classes; Calladine et al., 2018; Fuller & Robles, 2018; Hilmers et al., 2018). We also found that bird diversity increased again in mature age classes, although not to the same levels as the early successional stages. Overall, these results suggest that ecosystem functioning and resilience is lowest in the intermediate successional stages (thicket or pole classes). Beyond these broad trends, the results clearly show that different age classes have differing importance for the various diversity metrics. Bird species identity will also vary, with different species having strong associations with different age classes. Maintaining a variety of age classes across the forest landscape will therefore be important to promote the various facets of bird diversity. It is notable that open and early successional habitat was so important across all diversity metrics: it is crucial that management promotes these habitats, either through maintenance of permanent open areas or through periodic, rotational felling and re‐establishment of young trees in small areas.

The classic species–area relationship is well established (species richness increases with area) but the relationships between the various functional diversity metrics and area are more variable and less understood (Karadimou et al., 2016; Mazel et al., 2014; Wang et al., 2013; Zhang et al., 2021). In our study, we found that the total area variable explained a large proportion of the variance for four out of the six diversity metrics. Species richness and functional richness both increase with area, as expected given the species–area relationship and the commonly observed correlation between species and functional richness (MacArthur & Wilson, 1963; Oliveira et al., 2020; Pakeman, 2011; Smith et al., 2013). However, as found elsewhere, functional evenness somewhat counterintuitively decreased with area (Karadimou et al., 2016; Zhang et al., 2021). One explanation is that the total number of rare species recorded increases with area, resulting in an expanded area of trait space. However, individuals of common species are recorded at a faster rate than rare species. There is therefore both a larger trait space and an increasing skew between the proportion of individuals recorded in common versus rare species, so functional evenness decreases (Karadimou et al., 2016). Following this logic, we might also expect species evenness to decrease with area. However, this was not reflected in Shannon diversity (which combines species richness and evenness), which also showed a positive relationship with area. This could be because the species richness and area relationship was so strong or possibly functional evenness was affected to a greater extent than species evenness (for example, if increasing the survey area led to more rare species being recorded that had extreme trait characteristics).

Many forest bird species show a preference for either conifer or broadleaved trees, although habitat associations vary regionally (Felton et al., 2010; Roberge & Angelstam, 2006). Broadleaved forests generally tend to support a higher abundance and species richness than conifer forests, but this is variable (Archaux & Bakkaus, 2007; Felton et al., 2021). Mixed forests often have the greatest diversity because they can support both conifer and broadleaved specialists (Calladine et al., 2018; Fuller & Robles, 2018; Wesołowski et al., 2018). However, this is not a consistent relationship (Archaux & Bakkaus, 2007; Wesołowski et al., 2018) and in our study we found that management type was not a significant predictor for any diversity metric, despite being included in every final linear model. Generally, tree species composition affects the type and quality of resources that are available, whereas structural diversity is more important for providing shelter, nesting sites and safety from predators (Felton et al., 2021). Given that our analysis accounted for a wide range of other habitat and structural diversity metrics, our results suggest that tree species composition per se is less important than other structural characteristics (that provide resources such as shelter and nesting sites) in determining where birds spend most of their time. This is an important finding, adding to the debate surrounding whether plant composition or structure is more influential for predicting biodiversity (Adams & Matthews, 2019; Müller et al., 2010). Structure and floristics are related (Hewson et al., 2011), for example, even‐aged conifer plantations have lower structural diversity than mixed age natural broadleaved forests. These habitat variables therefore need careful disentangling to evaluate the causes of bird diversity patterns. Species‐specific responses to floristics versus structure will obviously differ. Of course, native broadleaved woodland habitats are highly valuable for biodiversity, and afforestation with native tree species is an important opportunity to expand this precious habitat. However, our results support the notion that predominantly coniferous plantations, which deliver other important resources such as timber, can also be important habitats for bird diversity, if habitat and structural diversity is encouraged in other ways (Graham et al., 2017).

Bird species associated with the shrub layer can be supported both by early successional stages in clear‐cut systems and by a shrub understorey in continuous‐cover systems (Calladine et al., 2015). Our finding that shrub density was a weak but significantly positive predictor of functional dispersion, despite relatively little overall variation in shrub density across the forest, highlights the importance of even subtle variations in shrub density for bird diversity, particularly in comparison to other structural attributes of forest stands. However, increasing shrub density can be problematic, particularly where herbivores are abundant. In the UK, there are extremely high densities of deer, including native red *Cervus elaphus* and roe *Capreolus capreolus* and nonnative fallow *Dama dama* and muntjac *Muntiacus reevesi*. This often results in a characteristic browse line where the understorey is removed; about 50% of British woodland has signs of herbivore browsing damage (Eichhorn et al., 2017; National Forest Inventory, 2020). Increasing shrub density therefore requires significant intervention to manage deer, either through costly exclusion (such as fencing) or direct population control at the landscape scale (Tew et al., 2021).

Evidence that increasing fine‐scale structural diversity will benefit biodiversity is mixed (Nolet et al., 2018). Our own analysis found relatively little effect of structural variables that would be expected to be important if biodiversity was responding to fine‐scale variability, such as in uneven‐aged systems. For example, compartment horizontal heterogeneity was not a statistically significant predictive variable for any diversity metric. Vertical evenness, canopy density, and gap fraction were not included in the final linear models due to collinearity with age class, so their statistical significance was not tested (with the exception of gap fraction being included for functional dispersion). Even so, it is notable that they were attributed very low levels of *R* ^2^ in the hierarchical partitioning (which can adequately handle the collinearity between variables), indicating that they had little explanatory power, for species richness, Shannon diversity, and functional richness. Interestingly, they were attributed slightly higher *R* ^2^ values for functional evenness, divergence, and dispersion (and gap fraction was a significantly positive predictive variable for functional dispersion), which suggests that fine‐scale structural diversity is more important in predicting the spread in abundances across traits and differentiation between niches. It is possible that greater variation for some variables (e.g., horizontal heterogeneity) would have increased their influence and we also note that variation was lost when structural variables were averaged when the compartments were grouped together to calculate the species diversity metrics. However, overall, these results suggest that birds are not responding as strongly to fine‐scale structural variation within the compartment as might be anticipated. Age class was generally a stronger predictor of bird diversity.

A number of recent studies have concluded that increasing wider landscape heterogeneity is essential to maximize biodiversity in managed forests, whereas focusing on increasing within‐compartment heterogeneity has limited benefits if the wider landscape is homogenous (Heinrichs et al., 2019; Penone et al., 2019; Schall et al., 2018, 2020). Although we did not measure the effects of the wider landscape directly in this study, our results offer some support for this hypothesis. The consistent importance of age class across diversity metrics, in combination with the lower importance of fine‐scale structural attributes, such as vertical evenness and horizontal heterogeneity, suggests that creating a matrix of age classes across the forest (i.e., between‐compartment diversity) would be more effective at maximizing bird diversity than focusing solely on within‐compartment diversity. In this context, it is important to emphasize that mixed ages and open space were included within our age class categories; we do not therefore advocate that forest management should be purely rotational forestry of even‐aged compartments rather it should also include uneven‐aged silviculture (and other habitats) within the forest matrix. Finer‐scale structural diversity was more important for some facets of bird diversity, particularly functional divergence and dispersion, and different taxonomic groups will respond to heterogeneity at different spatial granularity. However, creating a mosaic of forest stand habitats through a focus on diversity at the between‐stand scale rather than exclusively at the within‐stand scale, is likely to be most beneficial for biodiversity (Heidrich et al., 2020; Schall et al., 2018).

Species richness is the simplest diversity metric and has received the most attention in relating forest management to biodiversity (Lelli et al., 2019). However, different diversity metrics show different responses to management (Bae et al., 2018; Penone et al., 2019; Santini et al., 2017). Our analysis confirmed this important observation. For example, whereas total area was by far the most important metric for four of the six diversity metrics, it was the lowest ranked variable and non‐significant for both functional divergence and dispersion. The structural variables explained greater variation for functional diversity than taxonomic diversity metrics. Additionally, in comparing the diversity of different age classes, the mixed age classes had the highest species richness (and second highest functional richness) but the lowest functional evenness and divergence. Focusing on a single diversity metric might have given a skewed importance to a particular age class, whereas by comparing a wide range of diversity metrics it was clear that a diversity of age classes is important to promote different facets of diversity. These results emphasize the importance of considering multiple diversity metrics in order to understand how forest management affects diversity, particularly in addition to species richness, which, by itself, can give incomplete conclusions.

Airborne laser scanning is increasingly being used to quantify habitat structures and their relationship to biodiversity (Davies & Asner, 2014; Deere et al., 2020). LiDAR can generate structural information at extremely high resolutions but this does not necessarily improve model prediction accuracy. Our hierarchical partitioning analysis of different spatial resolutions showed that the “best” resolution varied for different structural metrics, clearly demonstrating the value of scale optimization to determine the granularity that best captures how species respond to different environmental variables (Mcgarigal et al., 2016). Interestingly, we found that species were responding to the environment at either fine (0.5 m) or coarse (5 or 10 m) resolutions, with no metric favoring the intermediate 2‐m resolution. Our results also indicate that some facets of species diversity respond to horizontal heterogeneity at a coarser scale than top canopy height or gap fraction. This suggests that species perceive large‐scale horizontal structural patterns in a broad way (i.e., responding to general changes in structure at the scale of small clearings or groups of trees rather than minor canopy variation) but are more responsive to canopy gaps or overall canopy height at finer scales.

This study was subject to a range of limitations; for example, we did not consider the distance to forest edge or the wider landscape context (Fuller & Robles, 2018). LiDAR data was collected around a month before the bird surveys commenced, with greater leaf cover expected during the bird surveys, which may affect the structural variables derived from the LiDAR. However, there is evidence to suggest that leaf‐off LiDAR data is superior to leaf‐on data in describing habitat structure for biodiversity assessment (Froidevaux et al., 2016). Unfortunately, the LiDAR data covered different areas of the forest in the two sampling periods (2015 and 2017), so we were unable to incorporate the effect of year into the statistical analysis. Nevertheless, given that there was only 2 years' difference between the two sampling years, there was unlikely to be a substantial effect of year across all bird species.

We used the scale of a forest compartment for data collection and analysis, which has great practical application as it is the scale at which management decisions are taken. However, the spatial granularity used may change the order of importance of different environmental variables (Bae et al., 2018; Stein et al., 2014). The compartments in Thetford Forest were relatively small, in contrast to some other forest rotational management systems around the world, so this caveat needs to be considered when applying the results elsewhere. Finally, these results and recommendations apply to bird diversity, rather than other important conservation goals such as maximizing abundances of forest specialists or rare species.

Approximately one‐third of the world's forests are managed as production forests; this rises to 37% of forests in the temperate zone, and 52% of European forests (FAO, 2016). This represents a hugely important resource in which sensitive management could lead to significant gains for bird diversity. Our results will be most relevant to other areas of northern Europe (a key area being Scandinavia, where production forestry constitutes 72% of forest area), with similar tree species choice and management regimes. However, we expect the principles to have much wider relevance. In particular, the distinction between within‐ and between‐stand diversity warrants further investigation.

## CONCLUSION

In summary, based on these results we recommend that managers of commercial plantations focus on creating a varied matrix of age classes across the landscape to maximize overall bird diversity. Despite using highly detailed structural vegetation data, we found that tree age class was a better predictive variable of bird diversity and was collinear with (and therefore encompassed) many other variables that were less important. This conclusion is encouraging in its simplicity. Clearly, total area across the landscape is also a key factor for diversity and so, given that wide‐scale woodland planting is an important policy objective, ideally afforestation should connect existing forest patches or create extensive new forest areas (on land with otherwise low conservation value). Within these large blocks, relatively small management compartments will facilitate species movement and resource utilization. Across forest landscapes, an approach that focuses on between‐stand diversity, through careful forest planning and timing of operations to maximize the variability in age classes (including mixed and open stands), is a simple and effective bird diversity conservation recommendation for practitioners.

## CONFLICT OF INTEREST

The authors declare no conflict of interest.

## Supporting information


Appendix S1

Appendix S2

Appendix S3

Appendix S4

Appendix S5

Appendix S6

Appendix S7

## Data Availability

Data (Tew et al., 2022) are available from the University of Cambridge repository service Apollo at https://doi.org/10.17863/CAM.83342.
