# Supplementary material for: Recommendations to enhance breeding bird diversity in managed plantation forests determined using LiDAR
Source: Ecol Appl. 2022 Aug 3;32(7):e2678. doi: 10.1002/eap.2678 (PMC9787994; doi:10.1002/eap.2678)
Supplement: Supplementary file 1 — Appendix S1 [file EAP-32-e2678-s007.pdf]

*Eleanor R. Tew, Greg J. Conway, Ian G. Henderson, David T. Milodowski, Tom Swinfield, William J. Sutherland. Recommendations to enhance breeding bird diversity in managed plantation forests determined using LiDAR. Ecological Applications.*

# Appendix S1

## Vertical plant area distribution profile calculation

For each 10-m square, the LiDAR point cloud was split into 1m vertical segments and the plant area distribution (PAD) calculated as the log of the fraction of points intercepted within the 1-m segment:

$$\mathrm{PAD_i} = \frac{1}{\Delta z}\ln\left(\frac{n(k = 1, z \le z_1)}{n(k = 1, z \le z_2)}\right)$$

Where, $z_1$ is the upper height (measured from the ground) of the vertical segment i, $z_2$ is the lower height (measured from the ground) of the vertical segment i, $\Delta z = |z_2 - z_1|$ (i.e. 1 as this was calculated for 1-m segments), $n(k = 1, z \le z_1)$ is the number of returns entering the top of the segment, $n(k = 1, z \le z_2)$ is the number of returns penetrating through the segment to lower vertical segments (Milodowski *et al.*, 2021). Ground returns pass all the way through the column, so they were not intercepted even within the lowest layer. This method assumes that vegetation is randomly distributed within each vertical segment (k = 1). Other studies deal with issues of canopy clumping by applying a scalar correction factor (k) to more accurately predict true leaf area density (Milodowski *et al.*, 2021; Stark *et al.*, 2012); however, this is an unknown constant and there are no available estimates from a similar managed forest system, so a scalar correction constant was not used. Applying a correction factor would not affect our conclusions unless a constant was applied differentially across the landscape, for example for different habitat types. The calculated plant area distribution values therefore represent an effective intercepting value rather than a true estimate.

For each forest compartment we extracted the mean plant area distribution values of a segment if $i \le I_{max}; I_{max} = \mathrm{round}[tch + (2 \times SD_{tch})]$, where i is the vertical segment, tch is the mean top canopy height for the compartment calculated at the 10m resolution (to match the resolution of the plant area distribution data), $SD_{tch}$ is the standard deviation of the top canopy height for the compartment at the 10 m resolution, and round indicates the value is rounded up to the nearest integer. This captured the majority of the variation in top canopy height across the compartment, and excluded any erroneous values from above the canopy as a result, for example, of bird hits in the LiDAR point cloud.

To inspect the plant area distribution profiles, we calculated the percentage of values that occurred in each vertical segment. We averaged these percentage values for each of the broad management type and age class combinations; the profiles are shown in Figure S1.

## Literature cited


Milodowski, D. T. *et al.* (2021) 'The impact of logging on vertical canopy structure across a gradient of tropical forest degradation intensity in Borneo', *Journal of Applied Ecology* 58: 1764–1775. doi: 10.1111/1365-2664.13895

Stark, S. C. *et al.* (2012) 'Amazon forest carbon dynamics predicted by profiles of canopy leaf area and light environment', *Ecology Letters* 15: 1406–1414. doi: 10.1111/j.1461-0248.2012.01864.x.

**a**

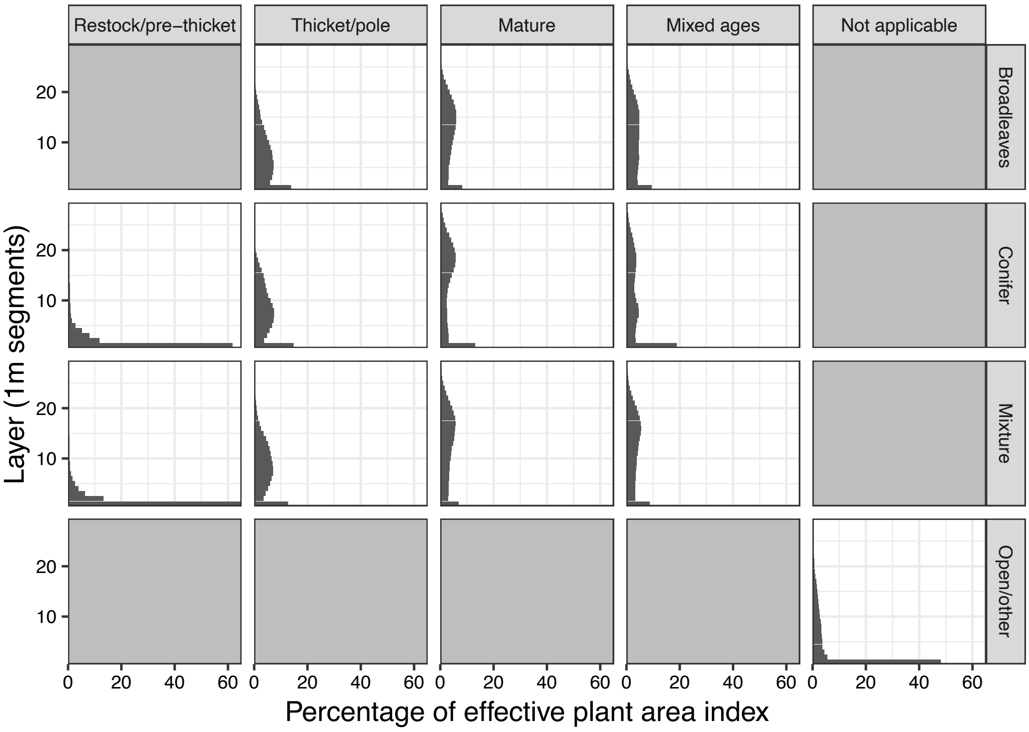


**b**

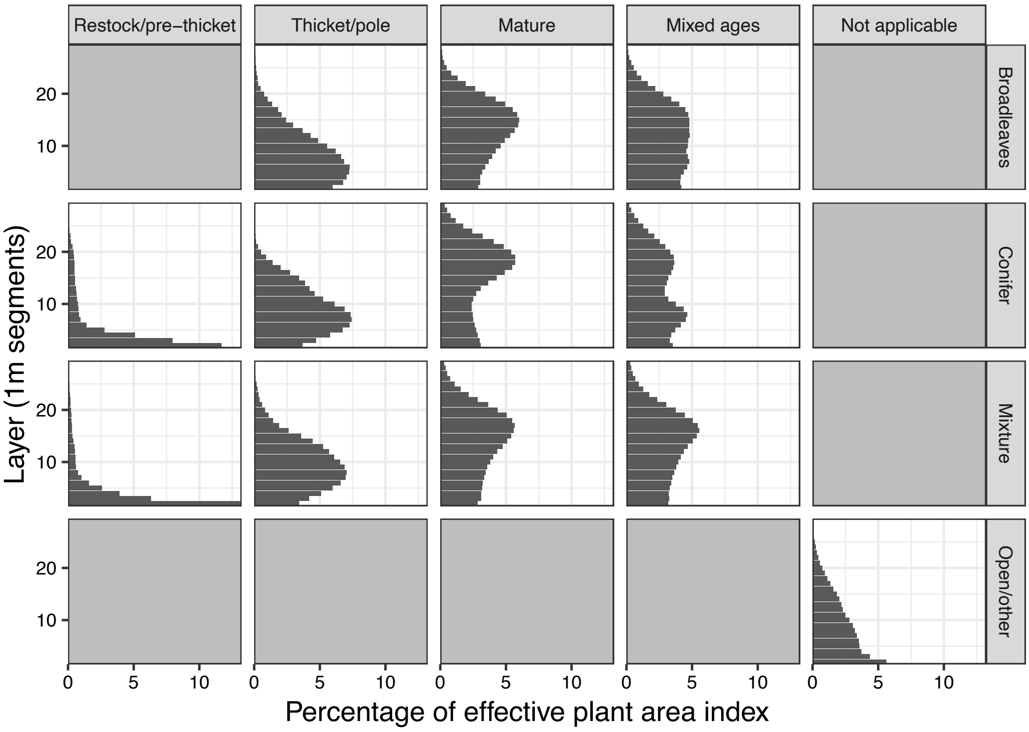


Figure S1: Plant area distributions (calculated as the percentage of the effective plant area index for each 1m vertical segment) for different types of forest stands. Boxes are greyed out where there were no data for these combinations used in the final analysis. a) All 1m vertical segments included. b) The first 1m layer (i.e. the ground layer) is excluded to show a smaller range of x-axis values and greater detail for the vegetation layers.
