# Supplementary material for: Recommendations to enhance breeding bird diversity in managed plantation forests determined using LiDAR
Source: Ecol Appl. 2022 Aug 3;32(7):e2678. doi: 10.1002/eap.2678 (PMC9787994; doi:10.1002/eap.2678)
Supplement: Supplementary file 2 — Appendix S2 [file EAP-32-e2678-s006.pdf]

Eleanor R. Tew, Greg J. Conway, Ian G. Henderson, David T. Milodowski, Tom Swinfield, William J. Sutherland. Recommendations to enhance breeding bird diversity in managed plantation forests determined using LiDAR. Ecological Applications.

Appendix S2

Number of compartments and total area in each of the 31 groups used in the statistical analysis

Values in brackets break down values by year as follows (2015; 2017). Grey shading indicates combinations that are not possible. Values of 0 indicate groups where there were not enough data to include in the analysis (i.e. only groups with >= 10 compartments were included).

Number of compartments

Simplified management category	Detailed management category	Restock/pre-thicket		Thicket/pole		Mature	Mixed	Not applicable	Total
		Restock	Pre-thicket	Thicket	Pole				
Conifer	Corsican pine monoculture	0	37 (37; 0)	181 (136; 45)	262 (213; 49)	92 (61; 31)	0		572
Conifer	Scots pine monoculture	20 (14; 6)	19 (17; 2)	20 (15; 5)	14 (14; 0)	150 (122; 28)	0		223
Conifer	Douglas fir monoculture	17 (15; 2)	0	0	0	10 (8; 2)	0		27
Conifer	Other conifer monoculture	14 (9; 5)	0	0	0	0	0		14
Conifer	Pure conifer mixture	0	12 (12; 0)	0	22 (17; 5)	61 (48; 13)	53 (45; 8)		148
Broadleaved	Birch monoculture	0	0	0	0	12 (8; 4)	0		12
Broadleaved	Other broadleaved monoculture	0	0	0	0	83 (62; 21)	0		83
Broadleaved	Pure broadleaved mixture	0	0	0	13 (11; 2)	76 (52; 24)	50 (35; 15)		139
Mixture	Mixture (primary	0	12 (8; 4)	0	18 (11; 7)	48 (42; 6)	107 (90; 17)		185

	component is conifer)								
Mixture	Mixture (primary component is broadleaved)	0	0	0	0	40<br>(36; 4)	74<br>(54; 20)		114
Open/other	Felled							27<br>(20; 7)	27
Open/other	Open							153<br>(126; 27)	153
Open/other	Open (with trees)							12<br>(11; 1)	12
Open/other	Other							43<br>(36; 7)	43
**Total**		51	80	201	329	572	284	235	1752

Total area (ha)

Simplified management category	Detailed management category	Restock/pre-thicket		Thicket/pole		Mature	Mixed	Not applicable	Total
		Restock	Pre-thicket	Thicket	Pole				
Conifer	Corsican pine monoculture	0	141.14<br>(141.14; 0)	581.7<br>(410.11; 171.59)	1121.19<br>(879.17; 242.02)	170.1<br>(104.93; 65.17)	0		2014.13
Conifer	Scots pine monoculture	51.07<br>(29.37; 21.7)	47.01<br>(40.66; 6.35)	35.46<br>(28.84; 6.62)	43.41<br>(43.41; 0)	181.24<br>(153.11; 28.14)	0		358.19
Conifer	Douglas fir monoculture	44.86<br>(40.54; 4.32)	0	0	0	24.39<br>(17.91; 6.49)	0		69.26
Conifer	Other conifer monoculture	20.94<br>(12.53; 8.41)	0	0	0	0	0		20.94
Conifer	Pure conifer mixture	0	16.01<br>(16.01; 0)	0	96.67<br>(63.92; 32.75)	114.09<br>(102.13; 11.96)	130.82<br>(105.63; 25.18)		357.58
Broadleaved	Birch monoculture	0	0	0	0	6.93<br>(5.37; 1.56)	0		6.93
Broadleaved	Other broadleaved monoculture	0	0	0	0	87.44<br>(70.3; 17.14)	0		87.44

Broadleaved	Pure broadleaved mixture	0	0	0	8.01<br>(7.53; 0.48)	95.61<br>(61.6; 34.01)	78.15<br>(53.6; 24.54)		181.77
Mixture	Mixture (primary component is conifer)	0	43.13<br>(27.66; 15.47)	0	55.51<br>(33.25; 22.25)	111.78<br>(106.05; 5.74)	294.55<br>(235.04; 59.51)		504.96
Mixture	Mixture (primary component is broadleaved)	0	0	0	0	60.52<br>(49.6; 10.91)	125.35<br>(103.82; 21.53)		185.87
Open/other	Felled							79.4<br>(72.79; 6.61)	79.4
Open/other	Open							252.78<br>(232.43; 20.34)	252.78
Open/other	Open (with trees)							15.42<br>(14.36; 1.06)	15.42
Open/other	Other							82.6<br>(70.92; 11.68)	82.6
**Total**		116.87	247.28	617.16	1324.78	867.52	628.87	414.78	4217.26
