# Supplementary material for: Recommendations to enhance breeding bird diversity in managed plantation forests determined using LiDAR
Source: Ecol Appl. 2022 Aug 3;32(7):e2678. doi: 10.1002/eap.2678 (PMC9787994; doi:10.1002/eap.2678)
Supplement: Supplementary file 3 — Appendix S3 [file EAP-32-e2678-s003.pdf]

Eleanor R. Tew, Greg J. Conway, Ian G. Henderson, David T. Milodowski, Tom Swinfield, William J. Sutherland. Recommendations to enhance breeding bird diversity in managed plantation forests determined using LiDAR. Ecological Applications.

Appendix S3

Trait database

Trait information was collated from various sources (see Table S1). Nest site individual categories are binary, foraging strata individual categories are estimated percentage use. Body mass, migratory status, nest site (individual categories – ground/cavities/other vegetation), diet category, foraging strata (individual categories – ground/understorey/mid-high vegetation/canopy/aerial) were included in the calculation of functional diversity metrics. Traits with more than one category were weighted accordingly (Laliberté and Legendre, 2010; Laliberté, Legendre and Shipley, 2014).

Species	Scientific name	Number of compartments with presence	Body mass (g)$	Migratory status$	Nest site+			Diet category*	Foraging strata*				
					Ground	Cavities	Other vegetation		Ground	Understorey (<2m)	Mid-high vegetation (>2m)	Canopy (in or from)	Aerial (well above vegetation)
Blackbird	Turdus merula	151	101.8	Resident	0	0	1	Omnivore	60	20	20	0	0
Blackcap	Sylvia atricapilla	358	17.7	Migrant	0	0	1	Omnivore	0	70	30	0	0
Blue Tit	Cyanistes caeruleus	264	10.9	Resident	0	1	0	Omnivore	10	30	30	30	0
Bullfinch	Pyrrhula pyrrhula	16	22.5	Resident	0	0	1	PlantSeed	0	50	50	0	0
Buzzard	Buteo buteo	23	828.2	Resident	0	0	1	VertFishScav	100	0	0	0	0
Carrion Crow	Corvus corone	193	508.7	Resident	0	1	1	VertFishScav	100	0	0	0	0
Chaffinch	Fringilla coelebs	448	21.8	Resident	0	0	1	Invertebrate	40	30	30	0	0
Chiffchaff	Phylloscopus collybita	332	7.7	Migrant	1	0	1	Invertebrate	25	25	25	25	0
Coal Tit	Periparus ater	376	9.1	Resident	0	1	0	Omnivore	0	0	20	80	0
Cuckoo	Cuculus canorus	30	114.3	Migrant	0	0	1	Invertebrate	20	20	40	20	0
Curlew	Numenius arquata	1	783.9	Partial/short distance migrant	1	0	0	Omnivore	100	0	0	0	0

Dunnock	Prunella modularis	60	21.2	Resident	0	0	1	Omnivore	100	0	0	0	0
Firecrest	Regulus ignicapilla	15	5.4	Partial/short distance migrant	0	0	1	Invertebrate	0	50	50	0	0
Garden Warbler	Sylvia borin	63	17.2	Migrant	0	0	1	Omnivore	0	60	20	20	0
Goldcrest	Regulus regulus	311	5.3	Partial/short distance migrant	0	0	1	Invertebrate	0	50	50	0	0
Goldfinch	Carduelis carduelis	18	15.8	Partial/short distance migrant	0	0	1	PlantSeed	20	60	20	0	0
Great Spotted Woodpecker	Dendrocopos major	33	78.7	Resident	0	1	0	Omnivore	0	20	50	30	0
Great Tit	Parus major	225	18.6	Resident	0	1	0	Omnivore	0	20	60	20	0
Green Woodpecker	Picus viridis	26	188.9	Resident	0	1	0	Invertebrate	100	0	0	0	0
Greenfinch	Carduelis chloris	8	27.7	Resident	0	0	1	PlantSeed	40	30	30	0	0
Jackdaw	Corvus monedula	13	231.8	Resident	0	1	0	Omnivore	50	20	20	10	0
Jay	Garrulus glandarius	70	166.8	Resident	0	0	1	Omnivore	60	20	20	0	0
Kestrel	Falco tinnunculus	3	205.5	Resident	0	1	1	VertFishScav	80	10	10	0	0
Linnet	Carduelis cannabina	45	18.8	Migrant	0	0	1	PlantSeed	60	20	20	0	0
Long-tailed Tit	Aegithalos caudatus	86	7.8	Resident	0	0	1	Invertebrate	10	40	10	40	0
Magpie	Pica pica	26	212.9	Resident	0	0	1	VertFishScav	70	20	10	0	0
Marsh Tit	Poecile palustris	36	10.7	Resident	0	1	0	Omnivore	0	20	80	0	0
Meadow Pipit	Anthus pratensis	6	18.9	Partial/short distance migrant	1	0	0	Invertebrate	70	20	10	0	0
Mistle Thrush	Turdus viscivorus	24	125.9	Resident	0	0	1	Omnivore	60	20	10	10	0
Nuthatch	Sitta europaea	67	22.1	Resident	0	1	0	Invertebrate	33.3	33.3	33.3	0	0
Pheasant	Phasianus colchicus	110	1190.0	Resident	1	0	0	PlantSeed	100	0	0	0	0
Red-legged Partridge	Alectoris rufa	12	490.0	Resident	1	0	0	PlantSeed	100	0	0	0	0

Robin	Erithacus rubecula	514	19.0	Resident	1	0	1	Omnivore	50	50	0	0	0
Siskin	Carduelis spinus	53	12.9	Migrant	0	0	1	PlantSeed	0	50	50	0	0
Skylark	Alauda arvensis	27	38.6	Partial/short distance migrant	1	0	0	PlantSeed	100	0	0	0	0
Song Thrush	Turdus philomelos	179	75.0	Resident	0	0	1	Omnivore	100	0	0	0	0
Spotted Flycatcher	Muscicapa striata	4	14.5	Migrant	0	1	1	Invertebrate	0	0	40	60	0
Stock Dove	Columba oenas	16	326.3	Resident	0	1	0	PlantSeed	80	20	0	0	0
Stonechat	Saxicola torquatus	3	15.8	Partial/short distance migrant	1	0	1	Invertebrate	100	0	0	0	0
Tree Pipit	Anthus trivialis	45	21.6	Migrant	1	0	0	Invertebrate	100	0	0	0	0
Treecreeper	Certhia familiaris	71	8.8	Resident	0	1	1	Invertebrate	0	50	50	0	0
Turtle Dove	Streptopelia turtur	6	156.8	Migrant	0	0	1	Omnivore	100	0	0	0	0
Whitethroat	Sylvia communis	69	13.8	Migrant	0	0	1	Invertebrate	0	100	0	0	0
Willow Warbler	Phylloscopus trochilus	142	8.9	Migrant	1	0	1	Invertebrate	0	0	30	70	0
Woodlark	Lullula arborea	29	30.0	Partial/short distance migrant	1	0	0	Omnivore	100	0	0	0	0
Woodpigeon	Columba palumbus	403	507.4	Resident	0	0	1	PlantSeed	80	20	0	0	0
Wren	Troglodytes troglodytes	737	9.9	Resident	0	1	1	Invertebrate	50	50	0	0	0
Yellowhammer	Emberiza citrinella	117	25.9	Resident	1	0	1	PlantSeed	100	0	0	0	0

Table S1: Trait database.

*Data gathered from Elton Traits database (Wilman *et al.*, 2014). For nuthatch, we corrected values to be 33.3 instead of 33. Foraging strata status was refined for some species, for populations in Thetford Forest using local expert knowledge (Ian Henderson, Greg Conway, *pers. comm.*)

[+]Data gathered from Oliveira Hagen et al. (Oliveira Hagen *et al.*, 2017). We amalgamated the cavities in cliff, rocks, ground and tree cavities into one category 'cavities'. We removed the artefacts nest column as this database was created for the study of urban birds, and artificial structures are not generally present in the forest.

[$]Data gathered from BTO birdfacts (BTO birdfacts). For body mass, where there was great sexual dimorphism, the average was taken (curlews, pheasants). Migratory status was refined to the populations in Thetford Forest using local expert knowledge (Greg Conway, *pers. comm.*)

Literature cited

BTO birdfacts. Retrieved from https://www.bto.org/about-birds/birdfacts

Laliberté, E., & Legendre, P. (2010). A distance-based framework for measuring functional diversity from multiple traits. *Ecology*, *91*(1), 299–305. doi:10.1890/08-2244.1

Laliberté, E., Legendre, P., & Shipley, B. (2014). FD: measuring functional diversity from multiple traits, and other tools for functional ecology. R package version 1.0-12.

Oliveira Hagen, E., Hagen, O., Ibáñez-Álamo, J. D., Petchey, O. L., & Evans, K. L. (2017). Impacts of Urban Areas and Their Characteristics on Avian Functional Diversity. *Frontiers in Ecology and Evolution*, *5*, 84. doi:10.3389/fevo.2017.00084

Wilman, H., Belmaker, J., Simpson, J., De La Rosa, C., Rivadeneira, M., & Jetz, W. (2014). EltonTraits 1.0 : Species-level foraging attributes of the world's birds and mammals. *Ecology*, *95*, 2027.
