# Supplementary material for: Recommendations to enhance breeding bird diversity in managed plantation forests determined using LiDAR
Source: Ecol Appl. 2022 Aug 3;32(7):e2678. doi: 10.1002/eap.2678 (PMC9787994; doi:10.1002/eap.2678)
Supplement: Supplementary file 4 — Appendix S4 [file EAP-32-e2678-s002.pdf]

Eleanor R. Tew, Greg J. Conway, Ian G. Henderson, David T. Milodowski, Tom Swinfield, William J. Sutherland. *Recommendations to enhance breeding bird diversity in managed plantation forests determined using LiDAR. Ecological Applications.*

Appendix S4

Calculating the functional diversity metrics

Fig. S1 illustrates the calculation of four functional diversity metrics for a group of species with two continuous traits (Fig. S1a):

- Functional richness is the volume of functional space occupied by the community (the grey polygon in Fig. S1b).
- Functional evenness is a measure of the regularity of species abundances within the functional space (Fig. S1c). This is calculated by first determining the minimum spanning tree (connections between points that have the minimum total branch length) (Fig. S1c.1). The functional evenness index calculates the regularity of the species positions and abundances along this minimum spanning tree (Fig. S1c.2). Functional evenness increases as the distance between points or the distribution of abundances becomes more regular.
- Functional divergence is a measure of the distribution of abundances across functional space (Fig. S1d). Firstly, the centroid of all the points is found (black cross, Fig. S1d.1), and the distance to this centroid is then calculated for each point (grey dotted lines, Fig. S1d.1). The distances are averaged to find the mean distance value (black circle, Fig. S1d.2), and the abundance of each species point is then compared in relation to the position of the mean value (coloured lines, Fig. S1d.2 and Fig. S1d.3). If larger abundances are grouped below the mean value (below the line, Fig. S1d.3) and smaller abundances are found above the mean value (above the line, Fig. S1d.3), the functional divergence is low.
- Functional dispersion combines abundance with the distribution (i.e. volume) of species in functional space (Fig. S1e). Averaging the distances between each species point and the centroid of all species points (grey dashed lines, Fig. S1e.1) gives a description of species distribution. Weighting each species point by its abundance shifts the location of the centroid position (dark grey cross, Fig. S1e.2). Functional dispersion is the average distance between the new centroid and each species point, weighted by abundance.

Figure S1: Graphical explanations of the different functional diversity calculations. Graphs are based on figures in Villéger et al. (2008) and Laliberté and Legendre (2010). The coloured points in each panel represent a different species and point size corresponds to abundance. Axes titles from parts (b) onwards are the same as part (a). Note that in parts (b), (c.1), (d.1), (d.2) and (e.1), the species abundances are unimportant. Abundances are subsequently incorporated into calculations for functional evenness (c.2), functional divergence (d.3) and functional dispersion (e.2), as explained below. a) Scatter graph to show the relationship between two continuous functional traits. The following graphs show calculations of functional diversity for these hypothetical species. b) Functional richness is the volume of trait space occupied by the species, represented by the grey polygon. c) Functional evenness is a measure of the regularity of species and abundances across the trait space. c.1) The minimum spanning tree between the points is indicated by the grey interconnecting line. c.2) The minimum spanning tree is stretched linearly and scaled down. Functional evenness measures the regularity of the point positions and abundances. d) Functional divergence is a measure of the distribution of species abundance across trait space. d.1) The black cross indicates the centroid of all the points (excluding the influence of relative abundance). The grey dotted lines indicate the distance between each point and the centroid. d.2) The black circle indicates the mean distance between each point and the centroid. The coloured lines measure the deviation between each point and the black circle. d.3) The deviations are shown linearly. Functional divergence measures the abundances in relation to the black line (mean distance to the centroid), i.e. whether higher abundances are clustered above or below the line. e) Functional dispersion is a measure of the distribution (i.e. volume of trait space occupied) and abundances of species. e.1) The black cross shows the mean centroid point if all species had equal abundance. Note this is the same as part d.1. e.2) The mean centroid point is weighted by species abundances (dark grey cross; the black cross from (e.1) is shown in light grey for comparison). Functional dispersion is the mean distance between each point and the centroid (grey dashed lines), weighted by the species abundances.

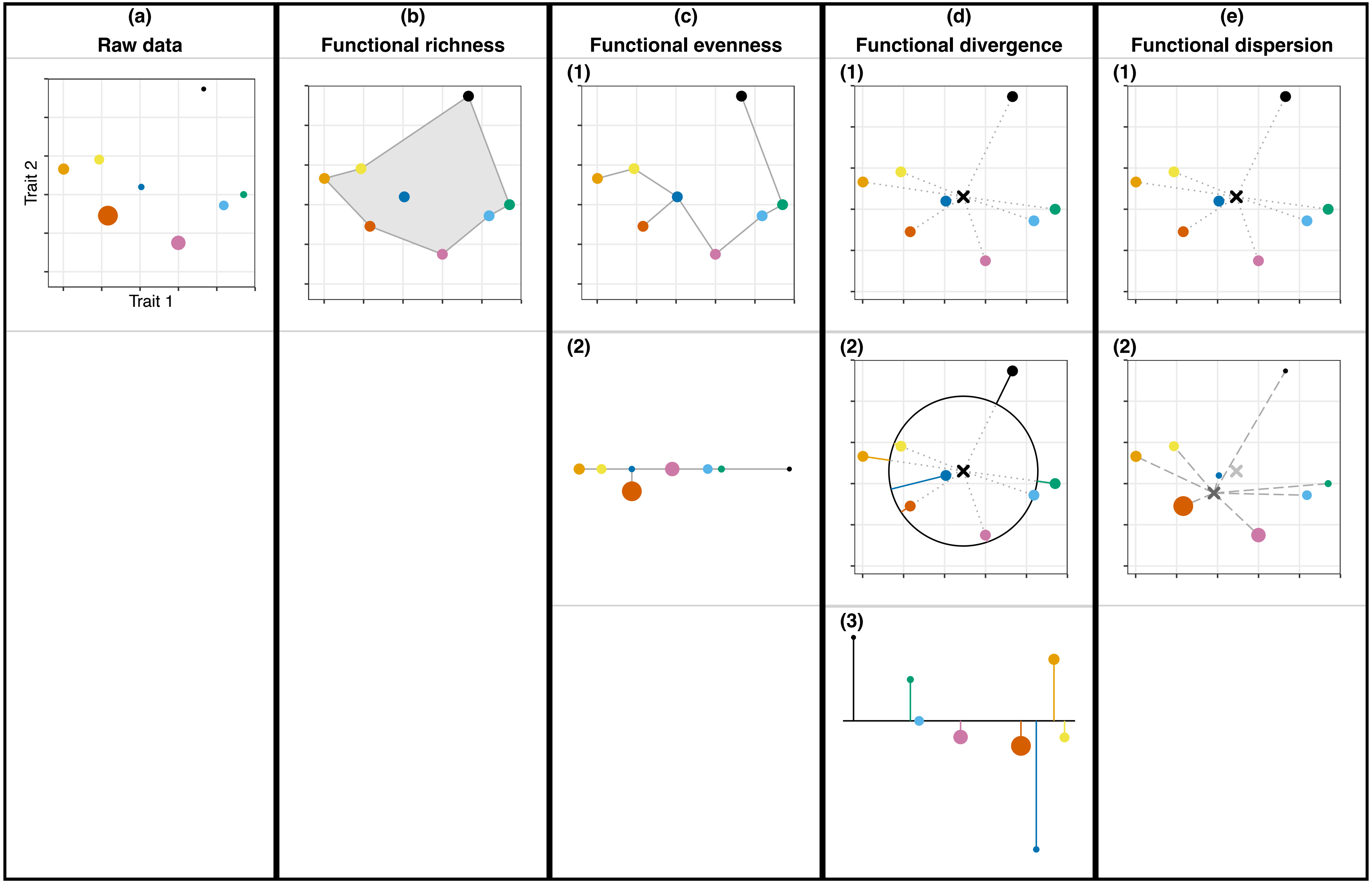
(a)
Raw data
Trait 2
Trait 1
(b)
Functional richness
(c)
Functional evenness
(1)
(2)
(d)
Functional divergence
(1)
(2)
(3)
(e)
Functional dispersion
(1)
(2)
