# Supplementary material for: Recommendations to enhance breeding bird diversity in managed plantation forests determined using LiDAR
Source: Ecol Appl. 2022 Aug 3;32(7):e2678. doi: 10.1002/eap.2678 (PMC9787994; doi:10.1002/eap.2678)
Supplement: Supplementary file 5 — Appendix S5 [file EAP-32-e2678-s005.pdf]

Eleanor R. Tew, Greg J. Conway, Ian G. Henderson, David T. Milodowski, Tom Swinfield, William J. Sutherland. Recommendations to enhance breeding bird diversity in managed plantation forests determined using LiDAR. Ecological Applications.

Appendix S5

Pairwise correlation tests between habitat variables

Tests of correlation between two variables were calculated as follows: Pearson's correlation between two continuous variables, R from the linear model between a continuous and categoric variable, Cramer's V between two categoric variables. Blue shading indicates values greater than |0.7|. Top canopy height, gap fraction, vertical evenness, canopy density and age class were all correlated to each other to a high degree.

		**Top canopy height**		**Horizontal heterogeneity**			**Gap fraction**			**Vertical evenness**	**Shrub density**	**Canopy density**	**Broad management type**	**Total area (log)**	**Age class**
		**0.5 m**	**5 m**	**0.5 m**	**5 m**	**10 m**	**0.5 m**	**5 m**	**10 m**						
**Top canopy height**	**0.5 m**		1.000	0.441	0.027	0.125	-0.858	-0.825	-0.831	0.761	-0.162	0.859	0.504	0.223	0.948
	**5 m**	1.000		0.440	0.026	0.125	-0.859	-0.825	-0.832	0.761	-0.163	0.860	0.504	0.225	0.948
**Horizontal heterogeneity**	**0.5 m**	0.441	0.440		0.614	0.095	-0.207	-0.129	-0.141	0.222	-0.413	0.154	0.620	-0.045	0.717
	**5 m**	0.027	0.026	0.614		0.510	-0.014	0.033	0.059	0.078	0.067	-0.161	0.560	0.018	0.452
	**10 m**	0.125	0.125	0.095	0.510		-0.327	-0.323	-0.312	0.260	0.349	0.308	0.331	0.420	0.309
**Gap fraction**	**0.5 m**	-0.858	-0.859	-0.207	-0.014	-0.327		0.994	0.991	-0.921	-0.266	-0.942	0.575	-0.262	0.919
	**5 m**	-0.825	-0.825	-0.129	0.033	-0.323	0.994		0.997	-0.936	-0.322	-0.934	0.551	-0.277	0.892
	**10 m**	-0.831	-0.832	-0.141	0.059	-0.312	0.991	0.997		-0.937	-0.286	-0.942	0.524	-0.286	0.900
**Vertical evenness**		0.761	0.761	0.222	0.078	0.260	-0.921	-0.936	-0.937		0.315	0.801	0.435	0.185	0.790
**Shrub density**		-0.162	-0.163	-0.413	0.067	0.349	-0.266	-0.322	-0.286	0.315		0.147	0.325	0.004	0.422
**Canopy density**		0.859	0.860	0.154	-0.161	0.308	-0.942	-0.934	-0.942	0.801	0.147		0.485	0.363	0.928
**Broad management type**		0.504	0.504	0.620	0.560	0.331	0.575	0.551	0.524	0.435	0.325	0.485		0.319	0.642
**Total area (log)**		0.223	0.225	-0.045	0.018	0.420	-0.262	-0.277	-0.286	0.185	0.004	0.363	0.319		0.330
**Age class**		0.948	0.948	0.717	0.452	0.309	0.919	0.892	0.900	0.790	0.422	0.928	0.642	0.330	
