# Supplementary material for: Recommendations to enhance breeding bird diversity in managed plantation forests determined using LiDAR
Source: Ecol Appl. 2022 Aug 3;32(7):e2678. doi: 10.1002/eap.2678 (PMC9787994; doi:10.1002/eap.2678)
Supplement: Supplementary file 7 — Appendix S7 [file EAP-32-e2678-s004.pdf]

Eleanor R. Tew, Greg J. Conway, Ian G. Henderson, David T. Milodowski, Tom Swinfield, William J. Sutherland. Recommendations to enhance breeding bird diversity in managed plantation forests determined using LiDAR. Ecological Applications.

Appendix S7

The resolutions used for each diversity metric.

Each variable was initially calculated at four different resolutions: 0.5, 2, 5 and 10 m. The 'best' resolution was selected using hierarchical partitioning to calculate the resolution that had the highest percentage of independent effects, for each combination of diversity metric and variable.

	**Variables with multiple resolutions**		
**Diversity metric**	**Top canopy height**	**Gap fraction (top third)**	**Horizontal heterogeneity**
Species richness	5m	5m	10m
Shannon diversity	5m	0.5m	5m
Functional richness	5m	5m	10m
Functional evenness	5m	10m	10m
Functional divergence	0.5m	0.5m	0.5m
Functional dispersion	0.5m	0.5m	0.5m
